# Screening and control of methicillin-resistant *Staphylococcus aureus *in 186 intensive care units: different situations and individual solutions

**DOI:** 10.1186/cc10571

**Published:** 2011-11-25

**Authors:** Anke Kohlenberg, Frank Schwab, Michael Behnke, Christine Geffers, Petra Gastmeier

**Affiliations:** 1 Institute for Medical Microbiology, Immunology and Hygiene, University of Cologne, Goldenfelsstrasse 19-21, 50935 Cologne, Germany; 2 Institute of Hygiene and Environmental Medicine, Charité University Medicine Berlin, Hindenburgdamm 27, 12203 Berlin, Germany; 3 National Reference Centre for Surveillance of Nosocomial Infections, Hindenburgdamm 27, 12203 Berlin, Germany

## Abstract

**Introduction:**

Controversy exists about the benefit of screening for prevention of methicillin-resistant *Staphylococcus aureus *(MRSA) in intensive care units (ICUs) and recent studies have shown conflicting results. The aim of this observational study was to describe and evaluate the association between MRSA incidence densities (IDs) and screening and control measures in ICUs participating in the German Nosocomial Infection Surveillance System.

**Methods:**

The surveillance module for multidrug-resistant bacteria collects data on MRSA cases in ICUs with the aim to provide a national reference and a tool for evaluation of infection control management. The median IDs of MRSA cases per 1000 patient-days (pd) with the interquartile range (IQR) were calculated from the pooled data of 186 ICUs and correlated with parameters derived from a detailed questionnaire regarding ICU structure, microbiological diagnostics and MRSA screening and control measures. The association between questionnaire results and MRSA cases was evaluated by generalized linear regression models.

**Results:**

One hundred eighty-six ICUs submitted data on MRSA cases for 2007 and 2008 and completed the questionnaire. During the period of analysis, 4935 MRSA cases occurred in these ICUs; of these, 3928 (79.6%) were imported and 1007 MRSA cases (20.4%) were ICU-acquired. Median MRSA IDs were 3.23 (IQR 1.24-5.73), 2.24 (IQR 0.63-4.30) and 0.64 (IQR 0.17-1.39) per 1000 pd for all cases, imported and ICU-acquired MRSA cases, respectively. MRSA IDs as well as implemented MRSA screening and control measures varied widely between ICUs. ICUs performing universal admission screening had significantly higher MRSA IDs than ICUs performing targeted or no screening. Separate regression models for ICUs with different screening strategies included the incidence of imported MRSA cases, the type of ICU, and the length of stay in independent association with the number of ICU-acquired MRSA cases.

**Conclusions:**

The analysis shows that MRSA IDs and structural parameters differ considerably between ICUs. In response, ICUs have combined screening and control measures in many ways to achieve various individual solutions. The incidence of imported MRSA cases might be helpful for consideration in the planning of MRSA control programmes.

## Introduction

Infections with methicillin-resistant *Staphylococcus aureus *(MRSA) are associated with increased mortality and excess costs [1,2]. Intensive care units (ICUs) are high-risk areas for the selection and transmission of multidrug-resistant bacteria [3,4], and surveillance data show that MRSA is endemic in ICUs in all German regions [5]. Measures to reduce MRSA transmission in ICUs include hand and environmental hygiene, contact isolation, and patient decolonization; however, the implementation of these control measures depends on the fast and reliable identification of MRSA carriers. The most effective strategies for MRSA screening are the subject of current research and discussion [6-10]. Active MRSA surveillance cultures have been shown to reduce MRSA infections not only in ICUs performing screening but also hospital-wide [11]; however, a recent review evaluating the effectiveness of active MRSA surveillance cultures in ICUs concluded that the amount and quality of existing evidence are not sufficient for definitive recommendations [12]. In addition, new laboratory tools that are improving and speeding up diagnostics continue to be developed [13], but their usefulness and cost-effectiveness have yet to be demonstrated [14].

Analysis of the influence of individual as well as structural risk factors and the impact of control efforts such as screening on MRSA transmission in hospitals is important for planning and evaluation of control programs. The aim of this observational study was to describe and evaluate the association of MRSA incidence densities (IDs) and screening and control measures in 186 ICUs participating in the German Nosocomial Infection Surveillance System. To achieve this aim, data on ICU structure and process parameters derived from a detailed questionnaire were correlated with the MRSA IDs of these ICUs from the years 2007 and 2008.

## Materials and methods

### Surveillance of MRSA cases

Detailed methods of the modules of the German Nosocomial Infection Surveillance System for surveillance of nosocomial infections and multidrug-resistant bacteria in ICUs have been described previously [5,15]. In short, the surveillance module for multidrug-resistant bacteria (MDR-KISS) collects, among other multidrug-resistant bacteria data, data on all admitted patients with MRSA, including colonized patients and patients with infections not fulfilling the definitions of nosocomial infections of the Centers for Disease Control and Prevention. Surveillance of MRSA started in 2003, and 240 ICUs regularly submitted data on MRSA cases at the beginning of this study in 2007. Surveillance is performed for all patients admitted to these ICUs; however, patient-based data are collected only for patients with a culture that was positive for MRSA and that was recovered during the ICU stay or for patients known to be carriers on admission. Cases are differentiated between imported and ICU-acquired on the basis of a temporal definition with a 48-hour interval between admission and detection of MRSA as a cutoff. The following data are collected for every patient carrying MRSA in participating ICUs: dates of admission, discharge, and first isolation of MRSA; import of MRSA (defined as known carriage or detection of MRSA in any clinical or surveillance culture within 48 hours of ICU admission) or acquisition during the ICU stay (defined as detection within more than 48 hours of ICU admission); presence of colonization or presence of an infection that requires treatment during the ICU stay; and, in case of infection, the site of infection. For the denominator, the total number of patient-days (pd) is determined without excluding the patient-days of MRSA-positive patients.

The data are collected and stored anonymously and according to national guidelines for data protection. All data collected by MDR-KISS and included in this study are obtained during routine surveillance required by the German Protection against Infection Act (*Infektionsschutzgesetz*) [16]. In paragraph 23 of this law, hospitals are obliged to continuously collect and analyze data on nosocomial infections and resistant pathogens. No additional data not covered by this law are used for this study. Ethical approval and informed consent are, thus, not required. Data are collected by trained medical or infection control personnel in individual ICUs and are submitted via a web-based surveillance portal to the central database of the national reference center. MRSA isolates of submitted cases are not collected for further analysis such as molecular typing. Mean and median IDs of MRSA cases per 1,000 pd with the interquartile ranges are calculated for all MRSA cases and the subcategories of imported and ICU-acquired cases from the pooled data of all ICUs as a reference for comparison with local MRSA IDs.

### Definitions of MRSA cases

1. MRSA case: any patient who is carrying MRSA and who is admitted to participating ICUs, including patients with infection and colonization as well as imported and ICU-acquired cases.

2. Imported MRSA case: a patient with known carriage of MRSA on admission or detection of MRSA in any clinical or surveillance culture within 48 hours of admission.

3. ICU-acquired MRSA case: a patient with detection of MRSA in any clinical or surveillance culture within more than 48 hours of admission.

### Survey of MRSA control measures

In 2008, a detailed questionnaire with 75 questions regarding ICU structure (for example, number of beds, single rooms, and health-care staff), microbiological diagnostics (for example, routine urine and respiratory surveillance cultures), MRSA screening procedures (for example, polymerase chain reaction-based or culture-based screening and patient population screened), MRSA control measures (for example, single-room isolation, pre-emptive isolation, and decolonization protocols), infection control education, and implementation of surveillance was sent to participating ICUs. Questions were directed at control practices performed in 2007. All ICUs participating in the MDR-KISS surveillance network were asked to complete the questionnaire; ICUs were not chosen prospectively for performing or not performing screening. Answers to the electronic questionnaire were submitted online to the national reference center.

To describe the use of combinations or bundles of control measures, a tree diagram, including the three key control parameters of screening policy, isolation of MRSA carriers, and decolonization, was constructed. For this diagram, three categories of screening (universal admission screening, targeted screening, and no screening) and three categories of isolation practices were distinguished. The categories for isolation practices were strict single-room isolation defined as isolation of all MRSA carriers in single rooms; single-room isolation or contact precautions defined as isolation of MRSA carriers in single rooms if possible (but if no single room was available, contact precautions in larger rooms were performed); contact precautions defined as performance of contact precautions for MRSA carriers in larger rooms and no attempt of single-room isolation. Median IDs of MRSA cases per 1,000 pd with interquartile ranges were calculated for nine subgroups of ICUs implementing different screening and isolation strategies.

### Generalized linear models

Generalized linear models (GLMs) were used to estimate the association between parameters derived from the questionnaire and the number of all MRSA cases and the number of ICU-acquired MRSA cases, respectively. In the analysis, we considered the three key control parameters described above and their interactions, pre-emptive isolation, and the structural parameters of hospital type and size and ICU type and size, short admissions of less than 48 hours of less than one third of admitted patients, the percentage of ventilator beds, the number or percentage of single rooms, and the nurse-to-patient ratio. The nurse-to-patient ratio was calculated as the number of nurses per shift per patient. For employed nurses in 2007, a 40-hour week, not including sick or vacation days, was considered.

Additionally, to adjust better for the severity of disease, the length of stay and device use (use of central venous catheters and ventilation per 100 pd) were considered as potential confounding parameters in the model. We used negative binomial distribution in the model instead of Poisson distribution because the variance exceeds the mean and we observed overdispersion for the number of MRSA cases. The log number of patient-days was treated as an offset in the model. A stepwise forward approach was applied for the multivariate analysis. Selection criteria were the highest chi-square value and a *P *value of less than 0.05 in the type III score statistic. The Akaike information criterion was used as goodness-of-fit measure in the GLM. *P *values of less than 0.05 were considered significant. All analyses were performed with SPSS (IBM SPSS Statistics; IBM Corporation, Armonk, NY, USA) and SAS (SAS Institute Inc., Cary, NC, USA).

## Results

### Surveillance of MRSA cases

Two hundred forty ICU-KISS ICUs with at least 1 month of MDR-KISS data in the period from 2003 to 2007 were asked to complete the questionnaire, and 186 ICUs responded (response rate of 77.5%). There was no significant difference by hospital type and size or ICU type and size between responding and non-responding ICUs. The 186 responding ICUs admitted 302,524 patients with 1,075,364 pd and 430,685 ventilator-days from 2007 to 2008. Four thousand nine hundred thirty-five MRSA cases occurred in these ICUs during the period of analysis; of these, 3,928 (79.6%) were imported and 1,007 MRSA cases (20.4%) were ICU-acquired according to the definitions. Calculated per ICU, this translates to a median of 9 MRSA cases (7 imported and 2 acquired) per year with a range from 0 to 163 yearly MRSA cases. Structural characteristics as well as device use and the IDs of MRSA cases in the 186 study ICUs are shown in Table 1.

**Table 1:** Structural characteristics, use of invasive devices, and incidence densities of methicillin-resistant *Staphylococcus aureus *in 186 intensive care units

Characteristics of ICUs	Responding ICUs
Type of ICU, number (percentage)	
Medical-surgical ICUs	88 (47.3)
Surgical ICUs	44 (23.7)
Medical ICUs	42 (22.6)
Other ICUs	12 (6.4)
Hospital type, number (percentage)	
University hospital ICUs	36 (19.4)
Teaching hospital ICUs	89 (47.8)
Non-academic hospital ICUs	61 (32.8)
ICU structure	
Number of beds, median (IQR)	11 (8-14)
Number of single rooms, median (IQR)	2 (1-4)
Percentage of ventilator beds, median (IQR)	76 (50-100)
Length of stay in days, median (IQR)	3.7 (2.8-4.8)
Nurse-to-patient ratio^a ^(IQR)	0.66 (0.56-0.76)
ICUs with less than 1/3 of short admissions of less than 48 hours, number (percentage)	59 (31.7)
Use of invasive devices, median (IQR)	
Ventilation use^b^	35.7 (23.5-49.5)
Central venous catheter use^b^	66.6 (52.6-80.0)
Urinary tract catheter use^b^	82.0 (72.8-90.1)
MRSA IDs, median (IQR)	
ID of all MRSA cases	3.23 (1.24-5.73)
ID of imported MRSA cases	2.24 (0.63-4.30)
Incidence of imported MRSA cases	0.79 (0.23-1.88)
ID of ICU-acquired MRSA cases	0.64 (0.17-1.39)

^a^Calculated by nurses per shift per patient; for employed nurses in 2007, a 40-hour week, not including sick and vacation days, was considered. ^b^Per 100 patient-days. ICU, intensive care unit; ID, incidence density per 1,000 patient-days; IQR, interquartile range; MRSA, methicillin-resistant *Staphylococcus aureus*.

### Survey of MRSA control measures

**Figure 1:**
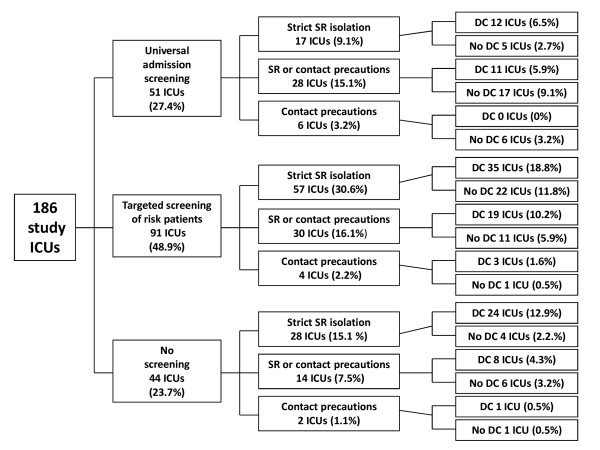
**Tree diagram of combinations of the three key control measures implemented in 186 intensive care units in this study**. The control measures are screening for methicillin-resistant *Staphylococcus aureus *(MRSA), isolation of identified MRSA carriers, and decolonization. DC, decolonization of all MRSA carriers; ICU, intensive care unit; SR, single room.

The results of the questionnaire for the most relevant MRSA screening and control measures are shown in Table 2. Individual MRSA control measures were implemented in several different combinations. Taking into account the three key control parameters of screening policy, isolation of identified carriers, and decolonization resulted in 17 different combinations (Figure 1). The most common combination of control measures used by ICUs was targeted screening, strict single-room isolation, and decolonization with mupirocin (Figure 1). The MRSA IDs of the 186 ICUs stratified by the implemented screening and isolation measures are shown in Table 3. The MRSA IDs of the different groups of ICUs varied significantly for all MRSA cases and imported MRSA cases but not for ICU-acquired MRSA cases (Table 3). A comparison of ICUs performing universal admission screening with ICUs performing targeted or no screening revealed that ICUs with universal admission screening had a significantly higher number of ICU and hospital beds, longer length of stay, and higher device use than ICUs with other screening policies (data not shown).

**Table 2:** Screening and control measures for methicillin-resistant *Staphylococcus aureus *in 186 intensive care units

MRSA screening and control measures	**Responding ICUs**, number (percentage)
ICUs with MRSA data and questionnaire results	186 (100.0)
Screening policy (*n *= 186)	
No MRSA screening	44 (23.7)
Any type of MRSA screening	142 (76.3)
Universal admission screening of all patients	51 (27.4)
Targeted screening of risk populations	91 (48.9)
Screening of known carriers on readmission	67 (36.0)
Screening of contact patients	62 (33.3)
Screening of defined high-risk patients	53 (28.5)
Screening method (*n *= 142)	
Culture-based MRSA screening	74 (52.1)
PCR-based screening	11 (7.8)
Culture- and PCR-based screening	57 (40.1)
Microbiologic diagnostics (*n *= 186)	
External microbiology laboratory	99 (53.2)
In-house microbiology laboratory	77 (41.4)
Performance of respiratory surveillance cultures	68 (36.6)
Performance of urine surveillance cultures	49 (26.3)
MRSA alert system (*n *= 186)	
Computer-based MRSA alert system	134 (72.0)
Paper-based MRSA alert system	19 (10.2)
Isolation of known MRSA carriers (*n *= 186)	
Isolation of MRSA carriers in SRs	174 (93.5)
Strict SR isolation	102 (54.8)
SR isolation if available or contact precautions in larger rooms	72 (38.7)
Contact precautions (and no SR isolation)	12 (6.5)
Pre-emptive isolation (*n *= 186)	
Carriers on readmission	111 (59.7)
Contact patients	76 (40.9)
Defined high-risk patients	67 (36.0)
All admissions	13 (7.0)
Topical decolonization (*n *= 186)	
Decolonization of all MRSA carriers with mupirocin	113 (60.8)
Decolonization of selected MRSA carriers with mupirocin	60 (32.3)
Mupirocin in combination with antiseptic washing	165 (88.7)

ICU, intensive care unit; MRSA, methicillin-resistant *Staphylococcus aureus*; PCR, polymerase chain reaction; SR, single room.

**Table 3:** Numbers and incidence densities of methicillin-resistant *Staphylococcus aureus *in 186 intensive care units stratified according to screening and isolation measures

Control measures	ICUs	MRSA cases	ICU-acquired MRSA cases	Patients	Patient- days	ID of all MRSA cases^a^	ID of imported MRSA cases^a^	Incidence of imported MRSA cases^a^	ID of ICU-acquired MRSA cases
	Number	Number	Number	Number	Number	Median (IQR)	Median (IQR)	Median (IQR)	Median (IQR)
Universal admission screening and strict SR isolation	17	676	102	24,887	112,122	5.69 (3.04-6.83)	3.74 (1.39-6.07)	1.98 (0.73-2.65)	0.71 (0.28-1.28)
Universal admission screening and SR or contact precautions	28	1,230	191	43,419	182,859	5.91 (3.02-9.88)	4.2 (2.39-9.45)	2.8 (1.31-3.37)	0.84 (0.37-1.62)
Universal admission screening and contact precautions	6	472	68	7,916	37,097	8.81	6.72	4.93	1.67
Targeted screening and strict SR isolation	57	1,298	287	98,396	341,332	2.55 (1.05-5.6)	1.87 (0.56-4.18)	0.6 (0.22-1.21)	0.42 (0.14-1.12)
Targeted screening and SR or contact precautions	30	696	169	51,054	162,083	2.91 (1.01-5.68)	1.47 (0.22-3.88)	0.62 (0.09-1.33)	0.67 (0-1.34)
Targeted screening and contact precautions	4	102	29	8,221	33,850	3.69	2.4	0.98	0.61
No screening and strict SR isolation	28	260	91	46,999	128,413	1.44 (0.05-3.34)	0.68 (0-2.19)	0.18 (0-0.91)	0.62 (0-1.03)
No screening and SR or contact precautions	14	190	68	19,715	69,517	3.31 (1.62-4.11)	2.05 (0.28-3.49)	0.73 (0.1-1.09)	1.16 (0.47-1.4)
No screening and contact precautions	2	11	2	1,917	8,091	1.37	1.12	0.47	0.24

^a^Significant difference by Kruskal-Wallis test (*P *< 0.001). ICU, intensive care unit; ID, incidence density; IQR, interquartile range (shown only if n > 10); MRSA, methicillin-resistant *Staphylococcus aureus*; SR, single room.

### Generalized linear models

The total number of MRSA cases per ICU was independently associated with the MRSA screening policy and the size of the hospital in the GLM (Table 4). Analyses of factors associated with ICU-acquired MRSA cases in ICU subgroups stratified according to screening strategies show an independent association of the type of ICU and the incidence of imported MRSA cases in the model with 51 ICUs with universal admission screening (Table 5); an independent association of the type of ICU, the incidence of imported MRSA cases, and the length of stay in the model with 91 ICUs with targeted screening (Table 6); and an independent association of the incidence of imported MRSA cases in the model with 44 ICUs without screening (Table 7) with ICU-acquired MRSA cases, respectively.

**Table 4:** Results of the multivariable analysis for all cases of methicillin-resistant *Staphylococcus aureus *

ICU structure and process parameters	IRR (95% CI)	*P *value^a^
Type of screening		
No screening	1 = reference	≤0.001
Targeted screening of risk patients	1.46 (1.00-2.14)	
Universal admission screening	2.73 (1.78-4.18)	
Size of the hospital		
Hospital size not greater than the median	1 = reference	0.001
Hospital size greater than the median	1.66 (1.22-2.24)	
Not significant in the multivariable analysis		
Isolation (strict SR isolation/SR or contact precautions/contact precautions)		
Decolonization with mupirocin (decolonization of all/not all MRSA patients)		
Interactions: screening-isolation, screening-decolonization, isolation-decolonization		
Pre-emptive isolation		
Short admissions of less than 48 hours of less than 1/3 of admitted patients		
Nurse-to-patient ratio (not greater than the median/greater than the median)		
Percentage of ventilator beds (not greater than the median/greater than the median)		
Number and percentage of SRs (not greater than the median/greater than the median)		
Size of ICU (not greater than the median/greater than the median)		
Type of hospital (university/academic/other)		
Type of ICU (medical-surgical/medical/surgical/other)		
Length of stay (not greater than the median/greater than the median)		
Device use (CVC, ventilation) (not greater than the median/greater than the median)		

In the generalized linear regression model, the negative binomial distribution was used and the log number of patient-days was treated as an offset parameter. A stepwise forward approach was applied, and the selection criteria were the highest chi-square value and^a^*P *value of less than 0.05 in the type III score chi-square statistic. CI, confidence interval; CVC, central venous catheter; ICU, intensive care unit; IRR, incidence rate ratio; MRSA, methicillin-resistant *Staphylococcus aureus*; SR, single room.

**Table 5:** Results of the multivariable analysis for ICU-acquired MRSA-cases of 51 ICUs performing universal admission screening

ICU structure and process parameters	IRR (95% CI)	*P *value^a^
Type of ICU		
Medical-surgical ICU	1 = reference	0.004
Medical ICU	0.41 (0.23-0.73)	
Surgical ICU	0.47 (0.28-0.80)	
Other ICU	0.46 (0.16-1.34)	
Incidence of imported MRSA cases		
Incidence of imported cases not greater than the median	1 = reference	0.026
Incidence of imported cases greater than the median	2.08 (1.09-4.00)	
Not significant in the multivariable analysis		
Isolation (strict SR isolation/SR or contact precautions/contact precautions)		
Decolonization with mupirocin (decolonization of all/not all MRSA patients)		
Interaction: isolation-decolonization		
Pre-emptive isolation		
Short admissions of less than 48 hours of less than 1/3 of admitted patients		
Nurse-to-patient ratio (not greater than the median/greater than the median)		
Percentage of ventilator beds (not greater than the median/greater than the median)		
Number and percentage of SRs (not greater than the median/greater than the median)		
Size of hospital and ICU (not greater than the median/greater than the median)		
Type of hospital (university/academic/other)		
Length of stay (not greater than the median/greater than the median)		
Device use (CVC, ventilation) (not greater than the median/greater than the median)		

In the generalized linear regression model, the negative binomial distribution was used and the log number of patient-days was treated as an offset parameter. A stepwise forward approach was applied, and the selection criteria were the highest chi-square value and^a^*P *value of less than 0.05 in the type III score chi-square statistic. CI, confidence interval; CVC, central venous catheter; ICU, intensive care unit; IRR, incidence rate ratio; MRSA, methicillin-resistant *Staphylococcus aureus*; SR, single room.

**Table 6:** Results of the multivariable analysis for ICU-acquired MRSA cases of 91 ICUs performing targeted screening

ICU structure and process parameters	IRR (95% CI)	*P *value^a^
Type of ICU		
Medical-surgical ICU	1 = reference	< 0.001
Medical ICU	0.32 (0.17-0.59)	
Surgical ICU	1.31 (0.80-2.16)	
Other ICU	0.45 (0.11-1.79)	
Incidence of imported MRSA cases		
Incidence of imported cases not greater than the median	1 = reference	< 0.001
Incidence of imported cases greater than the median	2.63 (1.69-4.00)	
Length of stay		
Length of stay not greater than the median	1 = reference	0.041
Length of stay greater than the median	1.58 (1.02-2.47)	
Not significant in the multivariable analysis		
Isolation (strict SR isolation/SR or contact precautions/contact precautions)		
Decolonization with mupirocin (decolonization of all/not all MRSA patients)		
Interaction: isolation-decolonization		
Pre-emptive isolation		
Short admissions of less than 48 hours of less than 1/3 of admitted patients		
Nurse-to-patient ratio (not greater than the median/greater than the median)		
Percentage of ventilator beds (not greater than the median/greater than the median)		
Number and percentage of SRs (not greater than the median/greater than the median)		
Size of hospital and ICU (not greater than the median/greater than the median)		
Type of hospital (university/academic/other)		
Device use (CVC, ventilation) (not greater than the median/greater than the median)		

In the generalized linear regression model, the negative binomial distribution was used and the log number of patient-days was treated as an offset parameter. A stepwise forward approach was applied, and the selection criteria were the highest chi-square value and^a^*P *value of less than 0.05 in the type III score chi-square statistic. CI, confidence interval; CVC, central venous catheter; ICU, intensive care unit; IRR, incidence rate ratio; MRSA, methicillin-resistant *Staphylococcus aureus*; SR, single room.

**Table 7:** Results of the multivariable analysis for ICU-acquired MRSA cases of 44 ICUs performing no MRSA screening

ICU structure and process parameters	IRR (95% CI)	*P *value^a^
Incidence of imported MRSA cases		
Incidence of imported cases not greater than the median	1 = reference	0.003
Incidence of imported cases greater than the median	1.94 (1.25-3.02)	
Not significant in the multivariable analysis		
Isolation (strict SR isolation/SR or contact precautions/contact precautions)		
Decolonization with mupirocin (decolonization of all/not all MRSA patients)		
Pre-emptive isolation		
Short admissions of less than 48 hours of less than 1/3 of admitted patients		
Nurse-to-patient ratio (not greater than the median/greater than the median)		
Percentage of ventilator beds (not greater than the median/greater than the median)		
Number and percentage of SRs (not greater than the median/greater than the median)		
Size of hospital and ICU (not greater than the median/greater than the median)		
Type of hospital (university/academic/other)		
Type of ICU (medical-surgical, medical, surgical, other)		
Device use (CVC, ventilation) (not greater than the median/greater than the median)		
Length of stay (not greater than the median/greater than the median)		

In the generalized linear regression model, the negative binomial distribution was used and the log number of patient-days was treated as an offset parameter. A stepwise forward approach was applied, and the selection criteria were the highest chi-square value and^a^*P *value of less than 0.05 in the type III score chi-square statistic. CI, confidence interval; CVC, central venous catheter; ICU, intensive care unit; IRR, incidence rate ratio; MRSA, methicillin-resistant *Staphylococcus aureus*; SR, single room.

## Discussion

Controversy exists about the benefit of screening for preventing MRSA transmission in hospitals and the most effective methods and strategies for MRSA screening programs, and recent studies have shown conflicting results regarding the effect of MRSA screening and control interventions [6-10,17-20]. In the present study, we evaluated the MRSA IDs and influencing factors in 186 German ICUs with a focus on MRSA screening procedures. The descriptive analysis shows large variation between ICUs in MRSA IDs as well as in MRSA screening and control measures implemented in daily practice.

### Distribution of MRSA incidence densities

Although MRSA was shown to be endemic in ICUs of all German regions [5], the MRSA IDs in the analyzed ICUs are not uniform. The MRSA burden in ICUs ranges from ICUs with no cases within the two-year period of analysis to 30.1 MRSA cases per 1,000 pd. Previous studies have shown that the benefit to be expected from active surveillance cultures is dependent on the local reservoir of MRSA carriers [21]. The variation between the MRSA IDs of ICUs in this study suggests that the extent of control efforts needed and their potential benefit for patients will differ considerably between ICUs.

### Impact of MRSA screening strategies

A comparison of the MRSA IDs of the ICUs in our sample stratified according to screening strategies shows that ICUs performing universal admission screening have significantly higher MRSA IDs than ICUs with targeted or no MRSA screening. However, it remains unclear to which extent the high MRSA IDs in ICUs with universal screening programs are the cause (implementation of screening because of a perceived MRSA problem) or the effect (better detection of MRSA carriers) of MRSA screening. A comparison of structure and process parameters shows that ICUs performing universal screening are high-risk units for MRSA with higher use of invasive devices, longer length of stay, and a higher number of ICU and hospital beds than ICUs without universal screening programs. In addition, the regression analysis shows an independent association of the size of the hospital and screening policies with MRSA cases. The influence of the structural parameter and the distortion caused by screening suggests that the total number of MRSA cases is not a suitable parameter for benchmarking of ICUs.

### Variation of MRSA control measures

Substantial variation in MRSA control measures between European countries has been described [22]. Our study shows that the implemented control measures also differ considerably between ICUs within one country. The evidence for the effectiveness of individual components of MRSA control such as screening, isolation, and decolonization has been described as weak [23]. In addition, the wide range of implemented combinations of control measures in association with differences in hospital structure, patient population, and imported MRSA incidence might explain the difficulty to evaluate the benefit of MRSA screening programs. The variety of different MRSA situations in the 186 ICUs indicates that studies of specific MRSA control strategies performed in a single ICU will have very limited general applicability.

### Generalized linear models

Factors associated with ICU-acquired MRSA cases in the GLMs analyzing three subgroups of ICUs with different screening strategies include the type of ICU, the imported MRSA incidence, and the length of ICU stay. The impact of these factors has been shown before [24,25] and is also further investigated in a separate analysis of a subgroup of ICUs from our sample [26]. The models show no association between a single MRSA screening or control measure and ICU-acquired MRSA cases; however, screening and other control measures are unlikely to be successful as isolated measures. A decision-analytical model has shown that the combination of active surveillance and decolonization was more effective than active surveillance alone [27], but the importance of individual components of MRSA control programs and the minimum effective measures needed for MRSA control are not known [28]. We therefore aimed to assess the interactions of control measures; however, after stratification of ICUs into subgroups according to implemented screening strategies to minimize MRSA ID distortion due to screening, no combination of isolation and decolonization measures was found to be associated with ICU-acquired MRSA cases.

The incidence of imported MRSA cases was associated with ICU-acquired cases independently of the implemented screening strategy and the effort made to identify MRSA carriers. This result indicates that the incidence of imported MRSA cases could provide a hint for the extent of control measures needed in ICUs.

### Limitations

In spite of including combinations of relevant control strategies and adjusting for the imported incidence of MRSA and proxy measures for the admitted patient population and disease severity, our model will fall short of capturing the complexity of MRSA control in ICUs. Further limitations are that the performed association analysis does not allow any conclusions about causation and that it is based on survey results that have not been validated by observation. We did not collect data on other important MRSA control variables such as compliance with hand hygiene and used a temporal definition to differentiate between import and acquisition of MRSA, and this might lead to a misclassification of a number of cases. Molecular typing was not performed, and the number of MRSA cases attributable to epidemic MRSA strains, community-acquired MRSA, and livestock-associated MRSA in participating ICUs remains unclear; however, a low percentage of community-acquired and livestock-associated MRSA in MRSA isolates from German hospitals has been reported [29].

## Conclusions

Our analysis shows substantial variation of MRSA IDs and screening and control measures among 186 ICUs. The wide range of differences regarding ICU structure and patient population as well as regarding MRSA IDs and control strategies implemented in daily practice indicates that ICUs are unlikely to benefit from a universal standardized approach to MRSA control. Different combinations of screening and isolation measures can be implemented without being associated with higher IDs of ICU-acquired MRSA cases. It might be more useful, instead of attempting a general proof of the effectiveness of MRSA screening, to better define the circumstances under which MRSA screening programs are most effective.

## Key messages

• Intensive care units (ICUs) show large variation in their methicillin-resistant *Staphylococcus aureus *(MRSA) incidence densities.

• ICUs have implemented MRSA control measures in several different combinations.

• The overall number of MRSA cases is distorted by MRSA screening.

• The combination and interaction of control measures have to be taken into account.

• The imported MRSA incidence is associated with ICU-acquired MRSA cases independently of the implemented screening strategy.

## Abbreviations

GLM: generalized linear model; ICU: intensive care unit; ID: incidence density; MDR-KISS: surveillance module for multidrug-resistant bacteria of the German Nosocomial Infection Surveillance System; MRSA: methicillin-resistant *Staphylococcus aureus*; pd: patient-days.

## Competing interests

The authors declare that they have no competing interests.

## Authors' contributions

MB supervised the web-based platform for the surveillance module, programmed the electronic questionnaire, and managed the data collection. FS analyzed and interpreted the data and performed the statistical analysis. AK analyzed and interpreted data and drafted the manuscript. PG was responsible for the concept, design, and implementation of the MDR-KISS surveillance module and critically revised the manuscript. All authors read and approved the final manuscript.
